# Expansion of the cassava brown streak pandemic in Uganda revealed by annual field survey data for 2004 to 2017

**DOI:** 10.1038/s41597-019-0334-9

**Published:** 2019-12-18

**Authors:** Titus Alicai, Anna M. Szyniszewska, Christopher A. Omongo, Phillip Abidrabo, Geoffrey Okao-Okuja, Yona Baguma, Emmanuel Ogwok, Robert Kawuki, Williams Esuma, Fred Tairo, Anton Bua, James P. Legg, Richard O. J. H. Stutt, David Godding, Peter Sseruwagi, Joseph Ndunguru, Christopher A. Gilligan

**Affiliations:** 1 National Crops Resources Research Institute, Kampala, Uganda; 2 Department of Plant Sciences, University of Cambridge, Cambridge, United Kingdom; 3 Mikocheni Agricultural Research Institute, Dares Salaam, Tanzania; 4 International Institute of Tropical Agriculture, Dares Salaam, Tanzania; 5 Farming Data, Cambridge, United Kingdom

**Keywords:** Plant molecular biology, Developing world, Agriculture

## Abstract

Cassava brown streak disease (CBSD) is currently the most devastating cassava disease in eastern, central and southern Africa affecting a staple crop for over 700 million people on the continent. A major outbreak of CBSD in 2004 near Kampala rapidly spread across Uganda. In the following years, similar CBSD outbreaks were noted in countries across eastern and central Africa, and now the disease poses a threat to West Africa including Nigeria - the biggest cassava producer in the world. A comprehensive dataset with 7,627 locations, annually and consistently sampled between 2004 and 2017 was collated from historic paper and electronic records stored in Uganda. The survey comprises multiple variables including data for incidence and symptom severity of CBSD and abundance of the whitefly vector (*Bemisia tabaci*). This dataset provides a unique basis to characterize the epidemiology and dynamics of CBSD spread in order to inform disease surveillance and management. We also describe methods used to integrate and verify extensive field records for surveys typical of emerging epidemics in subsistence crops.

## Abstract (summary)

**Table:** 

Measurement(s)	occurrence • cassava brown streak disease • Bemisia tabaci • Observed Incidence
Technology Type(s)	digital curation
Factor Type(s)	geographic location • year • date • time • variety sampled • field size • intercrop • number of neighbouring fields • crop age • altitude
Sample Characteristic - Organism	Manihot esculenta
Sample Characteristic - Environment	cultivated environment
Sample Characteristic - Location	Africa

Machine-accessible metadata file describing the reported data: 10.6084/m9.figshare.11302982

## Background & Summary

Cassava (*Manihot esculenta*) is one of the most important staple crops in sub-Saharan Africa, providing nutrition for over 700 million inhabitants of the continent. Cassava production is constrained by a number of pests and pathogens, with cassava brown streak disease (CBSD) being one of the most devastating. It has been established that CBSD is caused by cassava brown streak virus, (CBSV) and Ugandan cassava brown streak virus, (UCBSV) both belonging to the genus *Ipomovirus* and family *Potyviridae*^1–4^. Both viruses are transmitted by an insect vector, *Bemisia tabaci* (whitefly), and by human-mediated vegetative propagation of infected planting stems^5^.

The most damaging effect of CBSD is induced root necrosis, causing yield losses up to 70% and making the root unmarketable and entirely inedible in the most susceptible varieties^6–8^. Since cassava is widely grown as a subsistence crop, yield losses due to CBSD threaten food security for millions of households^7^. In addition, the economic development of smallholder farmers and larger producers is constrained, with yield losses estimated to be more than 750 million US dollars^9,10^ annually across the worst affected countries in eastern Africa alone.

For nearly 70 years, CBSD has been endemic in the coastal cassava growing areas of Kenya, Tanzania, Malawi and Mozambique^6^. In 2004, CBSD occurrence at high incidence was first reported in Mukono district of central Uganda. About the same time, similar CBSD occurrences were observed across northern Tanzania and western Kenya, and over time in multiple eastern, central and southern African countries. This new epidemic in the Great Lakes region of East and Central Africa represents a major expansion of CBSD beyond the endemic zones in the coastal lowlands of eastern Africa^1,8,11–17^. The disease currently poses a major threat to cassava production in Central and West Africa including Nigeria, the world’s largest cassava producer. Due to the huge socio-economic damage that CBSD poses, it is crucial to understand its epidemiology in order to predict likely net rates of human-mediated and insect vector disease dispersal, and to identify effective strategies for disease control and management^18,19^.

Here we present temporally and spatially-resolved annual surveillance data for Uganda from the start of the CBSD pandemic in 2004 up to the end of 2017, excluding 2016 when no survey was taken. The dataset is the result of work conducted by the National Crops Resources Research Institute (NaCRRI), Namulonge, in Uganda supported by funding from the Ugandan government, external donors including the World Bank, the Bill & Melinda Gates Foundation and the Association for Strengthening Agricultural Research in Eastern and Central Africa (ASARECA). The dataset focuses on visual scores of disease symptoms and we do not distinguish between different CBSD causal virus species. While the core surveillance protocol for collecting the most critical epidemiological variables remained the same, there were some changes to data collection during the course of the survey period, notably in switching from in-field paper to digital recording as well as minor changes in the nature and details of some recorded variables. We explain how problems were overcome in reconciling and validating data collected from extensive, rigorous, multiyear surveys, evolving protocols with consequent risks of global positioning and transcriptional errors.

**Fig. 1:**
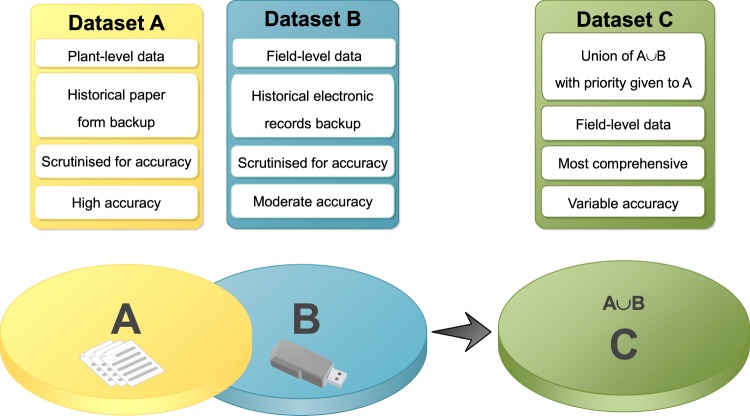
Venn diagram of Dataset C representing the union between Datasets A and B; C = A U B (with A having priority over B where duplicate records were identified).

We distinguish between two datasets according to the level of detail and validation that was possible for each dataset. Dataset A comprises a digitized dataset that has been fully validated against the original paper-format raw data records. These records additionally include plant-level information within all surveyed fields. Dataset B comprises historically digitized per-field summaries with variable and undocumented levels of scrutiny against the original data records. Dataset B is more extensive in some years with larger numbers of fields surveyed than Dataset A, but for which some of the original paper records are no longer available. We created a single unified Dataset C, by first summarizing plant level information from Dataset A into per-field summaries, and then adding to Dataset A those points from Dataset B not already represented in Dataset A (Fig. 1).

## Methods

### Sampling protocol

The surveys followed the sampling methodology outlined in Sseruwagi *et al*.^20^, originally developed for cassava mosaic disease (CMD) and adopted for CBSD surveys^21–23^. During each survey, cassava fields were visited at random along motorable roads in Uganda at intervals of 7–10 km. Intervals were extended up to 20 km in areas with limited densities of cassava fields. At each location a farmer was identified and asked for consent to survey the field. Fields with crops between 3–6 months after planting were selected for surveys, as CBSD foliar symptoms become apparent at this stage, and before leaf shedding. Field location coordinates were collected using handheld GPS devices and recorded either inside the field or in close proximity to its outer boundary, and as such field locations are considered accurate to within 100 meters. General field properties were recorded, including the approximate size of the field estimated by the surveyor, the cropping style (monoculture vs. intercrop), crop age (months since planting) estimated or obtained from the farmer, number of neighboring fields (within the accessible view of the surveyors), the name of the predominant cassava variety and the names of other cassava varieties that were present within the same field. In each field, regardless of its size, 30 plants of the predominant variety were surveyed along two diagonal transects, each of 15 plants, following an “X” pattern. Surveyors were advised not to actively select diseased plants and to choose plants uniformly at random within the transects instead. Each sampled plant was scored for severity of foliar and stem symptoms on a 1–5 scale^21–23^, where; 1 indicates no visible symptoms; 2 indicates mild vein yellowing or chlorotic blotches on some leaves; 3 indicates pronounced/extensive vein yellowing or chlorotic blotches on leaves (but no lesions or streaks on stems); 4 indicates pronounced/extensive vein yellowing or chlorotic blotches on leaves and mild lesions or streaks on stems; and 5 corresponds to pronounced/extensive vein yellowing or chlorotic blotches on leaves and severe lesions or streaks on stems, defoliation and dieback. Surveyors also recorded whether or not they saw CBSD in other varieties of cassava within the surveyed field or neighbouring fields. All surveyors participating in the surveys were trained in CBSD symptom recognition before actual surveys were undertaken. The cassava research team in Uganda has consistently collected disease survey data over multiple years since the 1990s, and has therefore accumulated a great deal of expertise and experience. The disease survey and sample collection protocol was reviewed, pre-tested and surveyors trained before every survey activity. The training focused on sample size, survey procedure, frequency of fields surveyed, disease parameters, symptom recognition and scoring, on which the surveyors were tested. Teams carried printed protocols to the field for reference with disease symptoms description and severity scoring scales included. In addition, field teams had senior scientists as team leaders whose key roles included daily on site cross-checking, review and validation of data collected by researchers under their supervision. The plant level assessment included counting and reporting the total number of adult whiteflies on the top five fully-expanded leaves of the tallest plant shoot. In the per-field summaries, a mean number of adult whiteflies identified on these top five leaves was recorded.

### Survey data acquisition - Datasets A and B

Between 2004 and 2015, data were recorded in the field on paper forms. Upon completing the survey, data from paper forms used in the field were transcribed by technicians into Excel spreadsheets as one row of summarized data per field, which included field properties information and average values for all numerical variables. These digital records were retrievable for 2008 to 2014, contain 4956 per-field records and comprise Dataset B.

More recently, all retrievable historic paper field survey forms were scanned and sent to a company specializing in digital data entry. Important elements such as locations, dates/times, plant severity scores and whitefly counts were digitized twice by two independent data entry operators and cross checked for accuracy. In 2017 plant-level data were recorded electronically, for the first time, with a custom form in the iForm app. The form was designed to ensure quality assurance with checks upon data entry, limiting the entry to a range of plausible values and formats. This included restricting entries to a specified range of integers for cassava severity scores (1–5) and whitefly count values (0–1000). Additionally, the use of a digital form allows for the presentation of more information on the screen, allowing for the inclusion of reminder prompts for the survey protocol such as explicitly displaying “Healthy” next to plant severity scores of 1. Upon syncing, the app data were immediately uploaded including per-plant and per-field level data for each surveyed field. These data comprise Dataset A which includes per-plant information collected from 4220 fields between 2004 and 2017, excluding years 2012 (no retrievable survey forms exist) and 2016 (no surveys were conducted).

Notably, one of the main differences between Dataset A and B is that they contain per-plant vs per-field data values respectively. Per-field summaries represent averages for any numeric variable recorded at plant level, including per-field disease incidence (proportion of infected plants). Severity means are the mean of diseased plants only. Thus, in infected fields, mean severity is the conditional average of severity scores for plants with a severity score above 1. If all 30 plants assessed in a field are free of CBSD symptoms, then the mean would be 1. Per-field summaries are a more limited representation of data. They lack representation of within-field variability of collected variables such as disease severity scores or adult whitefly numbers. Lack of per-plant data makes it harder to identify potential outliers or typographical errors.

### Union of Datasets A and B into Dataset C

**Fig. 2:**
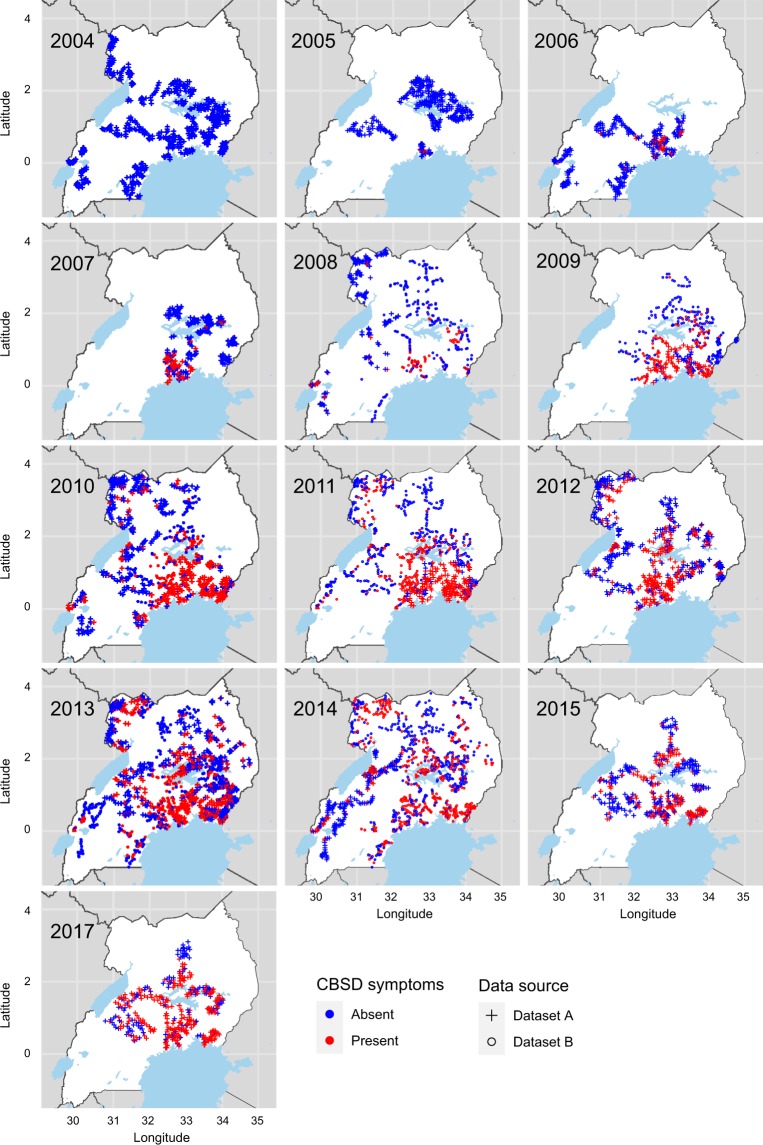
Dataset C field data collection locations. CBSD foliar symptoms are classified as present (red) or absent (blue). Dataset C is a union of the Dataset A (cross) that contains information at plant level in each field and is supplemented with additional information from the dataset B (circle) data.

A unified field level dataset, referred to as Dataset C, was derived by integrating the highly reliable digitised paper-based forms stored in Dataset A, with the field summaries from Dataset B (Fig. 1). Notably, Dataset B contained much larger numbers of recorded data points compared with Dataset A in years 2008–2014. We first summarized data from each survey field-level record in Dataset A and supplemented this information with remaining non-overlapping records from Dataset B. Therefore, where a field survey from Dataset A is identified in Dataset B, only the full, verified record from Dataset A is included in Dataset C (Table 1). Record matching between Datasets A and B was performed based on proximity of the spatial coordinates for each site and corresponding date of survey. Dataset C (Fig. 2) which encompasses 7627 field-summaries collected over 13 years provides the best achievable summary of historic CBSD status and spread in Uganda.

**Table 1:** Per-field summary data available for Uganda.

Year	2004	2005	2006	2007	2008	2009	2010	2011	2012	2013	2014	2015^a^	2017^b^	Total
Dataset A	600	476	300	253	189	105	514	200	—	606	200	374	403	4220
Dataset B	—	—	—	—	474	390	698	752	545	1066	1031	—	—	4956
Matched*	—	—	—	—	176	102	479	197	—	422	173	—	—	1549
Dataset C	600	476	300	253	487	393	733	755	545	1250	1058	374	403	7627

Dataset A represents quality-assured and highly accurate records digitized from retrievable paper-based forms by a specialist company, data digitized during a data entry workshop^a^, or collected with the iForm survey app in the field and downloaded from the server^b^. Dataset B represents previously typed survey records with most of the original paper forms no longer available. The resulting merged Dataset C represents the integration of two datasets with priority given to Dataset A where duplicate records were found, and supplemented with information from Dataset B for those records, where paper forms were not retrievable.

### Additional automatically generated covariates

**Fig. 3:**
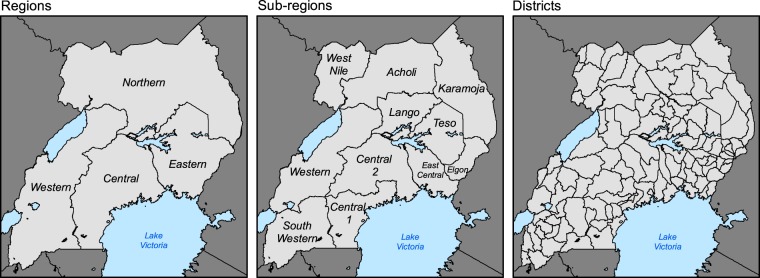
Three levels of administrative division of Uganda in 2014. Shapefiles are obtained from the Geo-Information Services Division, Uganda Bureau of Statistics.

Due to frequent transcription errors and multiple changes in administrative organization in Uganda over the survey period of 15 years, four columns with standardized administrative units were added to the Dataset A. Information about location within an administrative unit was derived using field geographic coordinates within a shapefile representing administrative units of Uganda, as of 2014, on four levels of hierarchical division: region, sub-region, district and county. The shapefiles were obtained from Geo-Information Services Division, Uganda Bureau of Statistics (Fig. 3). Altitude of the survey site was obtained from the digital elevation model (DEM) from the NASA Shuttle Radar Topography Mission (SRTM) version 4.1 at 90 m resolution (http://srtm.csi.cgiar.org/srtmdata/).

## Data Records

The datasets compiled in this study are recorded in English and within the geographic extent of Uganda. The data are freely available through the Figshare repository^24^, under the CC-BY-4.0 license waiver and we confirm that we have appropriate approval to share these data under this waiver. The data contains four files: DATASET_A.csv, DATASET_B.csv, DATASET_C.csv and LEGEND_CBSD_DATA.xlsx. DATASET_A.csv is organized in 126779 rows, each corresponding to a plant, where 30 plants are recorded in each field (except field id: ‘2015102’ which contains 29 plants records). DATASET_B.csv is organized into 4956 entries and represents per-field summary information recorded between 2008–2014.

DATASET_C.csv contains a total of 7627 per-field summary records. The origin of each data record (A or B) is stated in the ‘data_origin’ column and identified as ‘dbA’ or ‘dbB’ respectively. Records in this file can be linked to records in Datasets A or B based on two columns: ‘dbA_id’ and ‘dbB_id’ respectively. The csv files can be read with a wide variety of programs including Excel and R. The LEGEND_CBSD_DATA.xlsx explains the column headers in all three datasets files.

## Technical Validation

The following validation process applies to surveys between 2004 and 2015, for which data were recorded on paper forms and subsequently transcribed to a digital record. Surveys in 2017 were recorded digitally, hence requiring minimal additional validation. Here, we provide details of the procedures involved during validation.

### Coordinate cleaning

A Python program was written with approximately 30 different functions designed to parse 42 unique latitude notation patterns and 36 longitude patterns in the raw coordinate notation in Dataset A to decimal degrees. This program automated the conversion of 2924 coordinate records, 2581 of which were located within Uganda. The 350 parsed coordinates outside Uganda were commonly caused by transcription errors. For approximately 1000 remaining records, it was necessary to interpret the correct notation manually after visual inspection of the individual record. Visible outliers in Dataset B were manually rectified by identifying the same record in Dataset A. Subsequently, an R program was written to automatically plot the daily survey sites in both Dataset A and Dataset B to ensure that all records corresponded to a route that would be realistic in a single day. As required, the original survey form and neighboring surveys were used to identify errors and infer corrections based on the single and multi-day spatial sequence of survey sites.

### Variable cleaning

All variables were subjected to screening to ensure their formats and ranges of values were plausible in each column. For example, severity scores identified as 0, were converted to 1, to follow the overarching survey protocol^20^. Notably, field sizes were historically recorded in various formats including hectares, square meters, square kilometers and acres. Final field size outputs were converted to hectares. In some cases, the recorded units were difficult to infer and in those instances original values were retained, as the typical default value was hectares. In all columns with expected numeric outputs, in instances where additional text symbols appeared in the same entry, these letters and symbols were removed. Records where the values were not plausible were recorded as NA (Not Available).

### Validation against original paper forms

For key variables in Dataset A, a manual comparison with visual inspection was performed between the digitized record and the scanned survey form. Key variables are the field coordinates, survey date, field-level disease presence/absence, and adult whitefly counts. Coordinates were subjected to additional manual and automated validation in conjunction with the cleaning process described above.

Disease severity scores were in some cases not recorded on the paper forms if all plants scored 1 (i.e. no visible symptoms for disease) and thus resulted in a default record of NA (not applicable: interpreted as missing value) for all plants in a given field in Dataset A. We have identified those fields and recorded plant severity scores as 1 for all 30 plants. For survey fields in which values of 1 or higher were recorded for individual plants, in addition to NAs, we did not rectify missing values for plants in those fields. All variables were subjected to screening to ensure their formats and ranges of values were plausible in each category.

The cassava variety names were assessed based on visual judgement of trained field assistants or supplied by the farmer and were not genetically confirmed. The names of varieties vary locally and are not standardized in the form. In Dataset A those names might be additionally mistranscribed due to difficulty in reading local variety names.

### Verification levels

Different levels of verification were possible across variables in Dataset A and Dataset B (Table 2). Based on the level of verification possible, Table 2 highlights the verification level we have assigned to a given variable. Level 1 refers to a high degree of verification, involving both automated and manual values checks against paper forms. Level 2 refers to a medium level of verification, based largely on automated checks. Level 3 variables are unverified original transcriptions from paper forms. Level 4 variables were derived from external sources based on GPS surveyed field location coordinates. The administrative unit names were derived from shapefiles provided by the Geo-Information Services Division, Uganda Bureau of Statistics. Altitude (masl) was derived from the SRTM 90 meters digital elevation model (DEM) version 4.

**Table 2:** Variables collected in the survey with the distinction between variables derived from Datasets A and B, and collected at plant or field level.

Variable	Resolution	Verification level	
		Dataset A	Dataset B
date	Field	1	—
time	Field	2	—
year	Field	1	2
latitude, longitude	Field	1	2
village	Field	3	3
crop_age_months	Field	3	3
field_size_m2	Field	3	3
num_neighbouring_fields	Field	3	3
intercrop	Field	3	—
variety_sampled	Field	3	3
variety_2, variety_3, variety_4	Field	3	3
cbsd_foliar_presence cbsd_foliar_severity	Dataset A: Plant	1	—
cbsd_foliar_incidence cbsd_foliar_severity_mean	Dataset B: Field	—	2
cbsd_in_other_varieties	Field	3	3
adult_whitefly_count/ adult_whitefly_mean	Dataset A: Plant/Dataset B: Field	1	2
county, district, subregion, region	Field	4	3
altitude_masl	Field	4	3

Variables were subjected to a post-hoc verification process. 1 represents detailed post-hoc verification of variables using automated screening for plausible ranges of values and random manual checks. 2 represents moderate level of scrutiny ensuring variables are within a plausible range. 3 indicates lack of post-hoc verification process. 4 indicates the variable was derived from external sources based on GPS surveyed field location coordinates: administrative units were derived from shapefiles provided by the Geo-Information Services Division, Uganda Bureau of Statistics. Altitude (masl) was derived from the SRTM 90 meters digital elevation model (DEM) version 4.

### Error rates in Dataset B

**Fig. 4:**
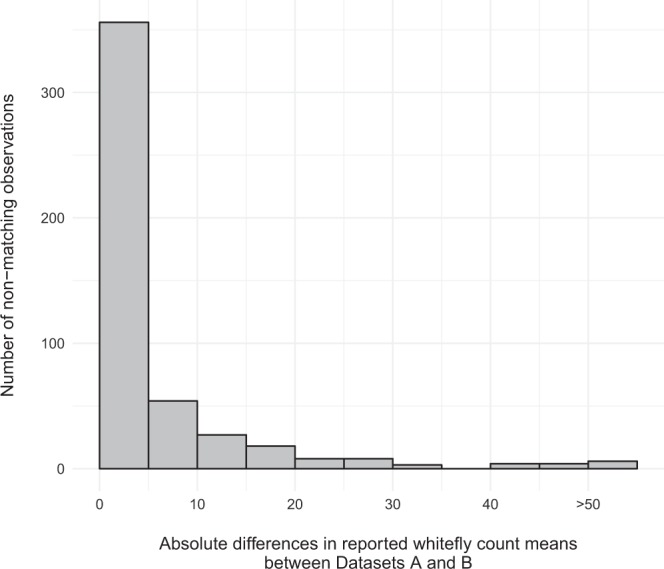
The distribution of deviations between matched records in Datasets A and B. A total of 488 out of 1549 (31.5%) matched field records had deviations in reported whitefly means higher than 0.09. Mean deviation = 4.389.

Records linked between both Dataset A and Dataset B were used to calculate the error rate of Dataset B according to incidence and disease presence/absence recording as well as whitefly numbers deviations. Dataset B incorrectly reported disease in 1.69% of corresponding fields that were recorded as healthy in Dataset A, and 5.61% of corresponding fields marked as diseased in Dataset A, were incorrectly reported as healthy in Dataset B. We also investigated the ratio of mean adult whitefly count records differing above an arbitrarily selected threshold of 0.09 for allowable tolerance, which is typically equivalent to a difference of 2 or less total reported whiteflies within the field (Fig. 4). Nearly one third of the matched records (31.5%) had deviations in mean whitefly numbers above the tolerance level. The largest proportion, however (19.2% of matched records), had minor deviations of 0.09 to 1 in the reported mean adult whitefly count. The remaining records had deviations of 1 to 5 (6.3%), 5 to 10 (2%), 10 to 25 (2.7%), 25 to 50 (1%) and above 50 (0.3%).

## Usage Notes

These datasets can be used to investigate the characteristics and changes in the spatio-temporal distribution of the CBSD pandemic in Uganda between 2004 and 2017 including, and not limited to, changes in disease prevalence, incidence, severity, density of the vector and varietal distribution. It is a highly valuable dataset from the epidemiological perspective and can be used to parameterize and test epidemiological spread distribution models, which can be applied to understand the rate of disease spread and areas of the highest risk of invasion outside the current pandemic zones in Africa and globally wherever cassava is grown. While the first CBSD case was reported in Uganda in November 2004^8^ at the demonstration fields at Mukono Zonal Agricultural Research and Development Institute (ZARDI), this detection was not part of the national surveys included in this dataset, which took place earlier in the year, between February and September. Thus, the first CBSD occurrence points appear only in 2005 in the attached dataset. We found that disease presence and absence data in Dataset B, after verification of the matched records with dataset A, have minor error rates that should be accounted for in the analysis. The false positive rate of CBSD presence is 0.97%, whilst the false negative rate was 2.39%. Nearly a third (31.5%) of adult whitefly count means have deviations in reported values greater than 0.09. Most of those discrepancies (19.2%) have minor deviations of 0.09 to 1 in reported whitefly count means per field. Thanks to unique record id’s these inconsistencies could be further explored and accounted for. We are unable to assess any variation in recorded variables that could stem from differences in the interpretation of field symptoms by individual field surveyors. Epidemiological models of CBSD spread trained on these data (to be reported elsewhere) enable exploration of disease control and management within affected area, and best management practices outside the current pandemic zone in preparation for when the disease arrives.

## Data Availability

The data cleaning, summarizing, merging and supplementing with additional columns were done in Python and R and the custom code used for this project can be provided upon request.
